# Analyzing Usage of the Metaverse by Associations of Patients With Prostate Cancer During the 2023 Blue Ribbon Campaign: Cross-Sectional Survey Study

**DOI:** 10.2196/63030

**Published:** 2025-05-13

**Authors:** Jung Ki Jo, Yeeun Kim, Yun-Sok Ha, Kwang Taek Kim, Sangjun Yoo, Woo Suk Choi, Jihye Yang, Jaeeun Shin, Sun Il Kim, Jeong Hyun Kim

**Affiliations:** 1 Department of Urology College of Medicine Hanyang University Seoul Republic of Korea; 2 Department of Medical and Digital Engineering Hanyang University Seoul Republic of Korea; 3 Department of Urology School of Medicine Kyungpook National University Daegu Republic of Korea; 4 Department of Urology Gachon University Gil Medical Center Incheon Republic of Korea; 5 Department of Urology Seoul Metropolitan Government - Seoul National University Boramae Medical Center Seoul Republic of Korea; 6 Department of Urology Konkuk University Medical Center Konkuk University School of Medicine Seoul Republic of Korea; 7 Enzaim Health Seoul Republic of Korea; 8 Department of Urology Ajou University School of Medicine Suwon Republic of Korea; 9 Department of Urology School of Medicine Kangwon National University Chuncheon Republic of Korea

**Keywords:** virtual reality, urology, prostatic neoplasm, self-help group, patient satisfaction, survey, questionnaire, digital health, prostate cancer, cancer, cross-sectional study, metaverse, medical education, patient education, patient engagement, technology, oncology, effectiveness, awareness, healthcare, urologic malignancy, morbidity, Korean

## Abstract

**Background:**

It is important to explain early diagnosis and treatment plans to patients of prostate cancer due to the different stages that diagnosis is made at and the corresponding stage-specific treatment options, as well as the varying prognoses depending on the choices made. Although various studies have implemented metaverse-based interventions across diverse clinical settings for medical education, there is a lack of publications addressing the implementation and validation of patient education using this technology.

**Objective:**

This study explored the potential of the metaverse as an educational and informational tool for prostate cancer. We measured and analyzed participants’ satisfaction and perceptions following a metaverse-based prostate cancer awareness campaign. We also evaluated the feasibility and potential effectiveness of the metaverse as a platform for hosting a virtual patient association and delivering health education.

**Methods:**

The study was conducted via a questionnaire administered from September 15 to October 20, 2023, during the Blue Ribbon Campaign organized by the Korean Urological Association and the Korean Society of Urological Oncology. The postevent questionnaire was designed to assess the effectiveness of using the metaverse to increase awareness of prostate cancer. A total of 119 participants, including patients, caregivers, and members of the general population, completed the survey within the metaverse space and assessed their satisfaction and perceived awareness using a 5-point Likert scale.

**Results:**

The mean educational satisfaction score was 4.17 (SD 0.65), the mean psychological satisfaction score was 4.06 (SD 0.70), the mean overall satisfaction score was 4.12 (SD 0.72), and the mean awareness score was 4.09 (SD 0.72) out of a possible 5 points. Among responses rated 4 or higher (“agree” or “strongly agree”), 82.8% (394/476) were in the educational aspect, 76.6% (365/476) in psychological satisfaction, 81% (289/357) in overall satisfaction, and 80.4% (287/357) in awareness. Statistical analysis revealed significant differences in psychological (median 4.0, IQR 3.50-4.63, vs median 4.50, IQR 4.0-4.56) and overall (median 4.0, IQR 3.67-4.83, vs median 4.33, IQR 4.0-4.67) aspects between the general population group and patients and caregivers (median 4.0, IQR 3.33-4.33, vs median 4.67, IQR 4.0-4.67).

**Conclusions:**

The findings suggest that the metaverse holds promise as a platform for health care education and patient support, offering accessible and engaging experiences for patients, caregivers, and members of the general population. Our approach demonstrated a positive influence on participants’ satisfaction and perceived awareness, highlighting its potential to enhance health communication and patient engagement. Despite these encouraging results, limitations, such as the sample being skewed toward younger participants and reliance on self-reported data, underscore the need for more rigorous and multidimensional assessment strategies. Future studies should incorporate objective knowledge assessments, behavioral follow-ups, and qualitative methods to better evaluate the intervention’s effectiveness. This study provides early evidence that metaverse-based interventions can support disease awareness and promote preventive health behaviors, contributing to the ongoing evolution of digital health education.

## Introduction

Prostate cancer is the second-most common cancer among men worldwide and the most common urologic malignancy in high-income countries. In 2020, there were an estimated 1,414,000 diagnosed cases and 375,304 deaths related to prostate cancer [1]. It is important to note that prostate cancer can progress without symptoms, leading to missed treatment opportunities [2]. It is characterized by a wide spectrum of diagnosis stages and treatments, ranging from low-risk stages with no treatment and active surveillance to high-risk stages treated with prostatectomy and various treatment modalities, such as radiation, hormone therapy, and chemotherapy. Early diagnosis and explanation of the treatment plan appropriate to the patient’s stage is critical, as treatment options and prognoses vary depending on stage [3,4].

To reduce morbidity and mortality associated with prostate cancer, it is important to accurately communicate knowledge and encourage early screening. In addition, patient awareness and active participation in the treatment process affect treatment outcomes and costs, so research on interventions to increase patient engagement is important [5,6]. Misconceptions need to be corrected, and reliable information needs to be communicated. Several studies have highlighted the importance of research to generate resources for the development and implementation of relevant educational programs [7,8].

To increase the effectiveness of prostate cancer education, patient association meetings were held in a metaverse space rather than in a traditional setting. Although there is no single definition of the metaverse, it is generally considered to be a superset of virtual reality and augmented reality and refers to a 3D virtual space that integrates physical and digital realities, where people can be represented virtually in a digital environment [9]. Gartner named the metaverse as one of its top 10 strategic technologies in 2019 and again in 2023, with a focus on accelerating strategies to capitalize on emerging virtual markets [10]. In 2022, Gartner also predicted that by 2026, 25% of people worldwide will spend at least 1 hour a day in the metaverse [11]. Use of the metaverse overcomes physical and time constraints and improves accessibility for both patients and health care providers and, once deployed, is repeatable. Additionally, there is no risk of spreading infectious diseases in virtual environments, ensuring safety, especially in pandemic situations, such as the recent COVID-19 outbreak [12,13]. A few previous studies have discussed the potential of metaverses [13-15] or focused on health care providers [16-18]. Among the studies that included the term ”metaverse” in searches of PubMed, Google Scholar, and Web of Science, no studies on patient association hosting and training were found, making it difficult to understand the potential effectiveness of metaverse-based cognitive improvement. In addition, relatively few studies have investigated educational uses of the metaverse [19].

The aim of this study was to measure participant satisfaction with and awareness of the metaverse as part of a worldwide prostate cancer awareness Blue Ribbon campaign. We hypothesized that satisfaction and awareness scores would vary by age, gender, relevance to patients, and geographic region.

## Methods

### Study Design

This national cross-sectional survey study aimed to evaluate participants’ satisfaction with and awareness of the Understanding the Prostate Cancer Metaverse event. A questionnaire was used to assess satisfaction with participation in the metaverse, and data were collected from September 15 to October 20, 2023, during the Blue Ribbon Campaign hosted by the Korean Urological Association and the Korean Urological Oncology Society. The study aimed to collect information about users’ awareness of their metaverse experience without imposing any restrictions on research participation.

### Participants

From September 15 to October 20, 2023, a total of 119 participants were recruited to participate in the 2023 Understanding the Prostate Cancer Metaverse event and responded to an online survey. Participants included male and female patients with prostate cancer (diagnosed or otherwise), caregivers, and individuals from the general population without any direct association to the disease or caregiving roles. As this study aimed to evaluate the feasibility of a novel digital intervention, no exclusion criteria were imposed and participation was voluntary. Recruitment was conducted through multiple online channels, including the official YouTube channel of the Korean Urological Oncology Society, prostate cancer–related communities, the Prostate Cancer Patient Advocacy Association, and online news articles.

### Procedures

**Figure 1:**
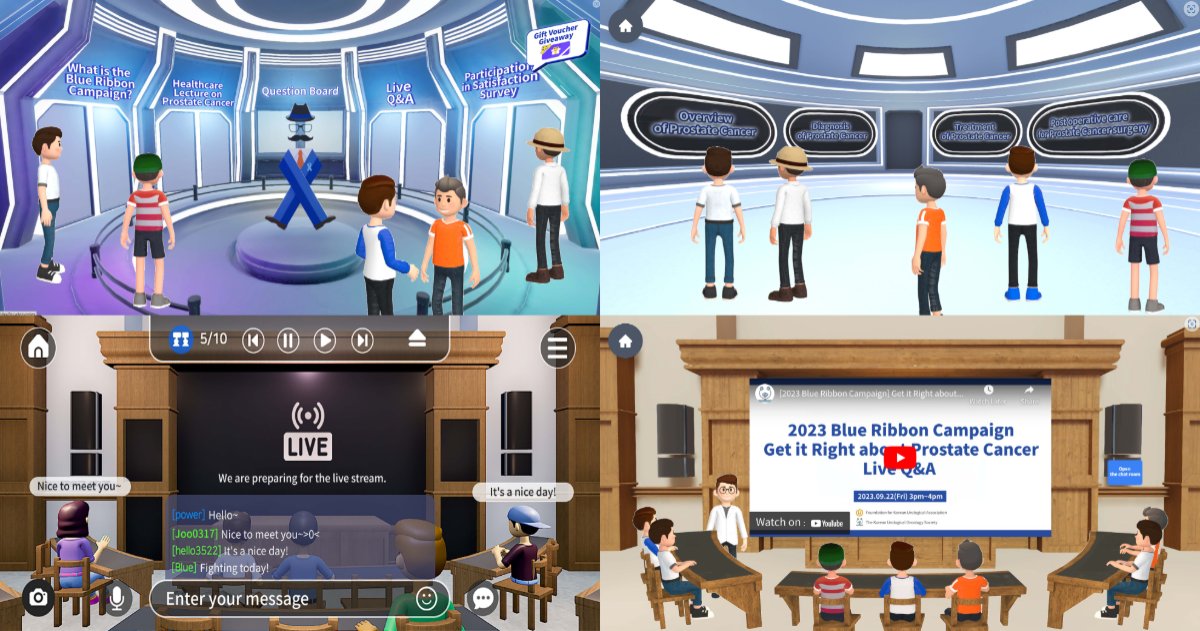
Schematic overview of the metaverse associations of patients with prostate cancer, showing (A) the campaign introduction area, (B) the informational sector, (C) a live Q&A session, and (D) a segment of an educational session. Q&A: question and answer.

The metaverse platform was developed with Caitory [20] for easy website and mobile access. Participants engaged with the event through online articles, communities related to prostate cancer, and YouTube. Within the metaverse environment, communication between clinicians and participants was facilitated via an on-screen chat interface. Health care lectures on prostate cancer, a question board, live question and answer (Q&A) sessions, and a room for participating in satisfaction surveys were established within the metaverse space. An overview of prostate cancer, along with diagnosis, treatment, and postoperative care, was offered as lecture topics for participants. The live Q&A session between clinicians and participants was recorded and made available for replay on YouTube. Figure 1 illustrates 4 distinct components designed to support participant engagement and health education. The campaign introduction area served as an entry space where users could learn about and interact with the campaign in a virtual reality setting (Figure 1A). The informational sector provided educational resources covering a range of prostate cancer topics, including risk factors, diagnostic methods, and treatment options (Figure 1B). Live Q&A sessions enabled real-time communication with experts, allowing users to submit health-related questions and receive responses during scheduled sessions (Figure 1C). Educational sessions featured a virtual classroom setting, where live lectures on health education were conducted, offering users an opportunity to listen to experts in real time and communicate directly in a virtual setting (Figure 1D).

### Online Survey

Distribution and collection of the questionnaire for this study were managed by Enzaim Health, a health care marketing communications company. The questionnaire design used the Delphi method to structure the questions through iterative feedback from a group of urologists. Finally, the questionnaire was reviewed and iteratively improved by members of the Korean Urological Oncology Society. Multimedia Appendix 1 presents the complete survey questionnaire. The questionnaire consisted of 20 mandatory items, with additional adaptive follow-up questions based on participant characteristics, such as gender, patient status, and prior experience with the metaverse. Consequently, participants answered between 18 and 22 questions, depending on the relevance of the items to their individual profiles (Multimedia Appendix 2). Some participants omitted answers that were not applicable. The questionnaire was divided into 4 parts: general information (8 questions), experience using the metaverse (2 questions), satisfaction with the Understanding the Prostate Cancer Metaverse event (7 questions), and awareness of using the metaverse in disease awareness programs (6 questions). Four of the questions in the survey used a 5-point Likert scale (from 1=strongly disagree to 5=strongly agree). A higher score on the scale indicated a more positive response. The remaining questions were presented as multiple-choice items.

The 4 items of the questionnaire used in this study were structured on a 5-point Likert scale and rated using the Cronbach α coefficient. Cronbach α for the total scale was .948, demonstrating excellent reliability, while each scale exhibited high internal consistency, with a minimum α value of .820, indicating reliable constructs.

### Statistical Analysis

Statistical analyses were performed using R version 4.3.2 (R Foundation for Statistical Computing) [21]. There were no missing data. Basic descriptive statistics were used to summarize each dataset. Cross-tests were analyzed using the chi-square test (*χ*²), while differences between groups were analyzed using the Kruskal-Wallis rank sum test and the Mann-Whitney U test as nonparametric tests. Significance was interpreted by reference to Bonferroni-corrected *P* values. The cutoff for the *P* value was .05. The reliability of the questionnaire was calculated using the Cronbach α coefficient [22]. To examine the relationship between the 4 scales measuring satisfaction with metaverse events, the Spearman correlation coefficient (ρ) was computed. Confirmatory factor analysis (CFA) was conducted to validate the predefined 4-factor structure of the questionnaire. The model fit was assessed using chi-square test, the comparative fit index (CFI), the Tucker-Lewis index (TLI), the root mean square error of approximation (RMSEA), and the standardized root mean square residual (SRMR). Based on established criteria, a model fit was considered acceptable if the CFI and the TLI were greater than 0.90, the RMSEA was below 0.08, and the SRMR was below 0.08. To assess convergent validity, the average variance extracted (AVE) and composite reliability (CR) were computed. A factor was considered to have adequate convergent validity if the AVE exceeded 0.50 and the CR was greater than 0.70. The results supported the construct validity of the measurement instrument, confirming that the questionnaire items adequately measure the intended latent constructs [23,24].

### Ethics Approval

The study adhered to STROBE (Strengthening the Reporting of Observational Studies in Epidemiology) guidelines, and all procedures were approved by the Institutional Review Board of Hanyang University Hospital (IRB file number: 2024-01-029). Participation was voluntary. All participants received the same standardized information through pre-recorded content and expert-led real-time interaction. Information about consent was included on the last page of the questionnaire. The purpose of the study and data confidentiality were clearly specified on the first page of the questionnaire and explained to all participants, and written informed consent was obtained from them before they participated in the survey (Multimedia Appendix 3). All content was processed to ensure that specific individuals could not be identified, in accordance with the Personal Information Protection Act. An incentive was provided by awarding gift certificates to 33 individuals through a random draw. Specifically, participants who completed the entire survey were entered into a prize draw to receive gift certificates, with KRW $50,000 (US $35.78) awarded to 3 winners and KRW $10,000 (US $7.16) awarded to 30 winners.

## Results

### Participant Characteristics

The age composition of the participants was notably skewed toward younger adults, with individuals in their twenties accounting for 37% (n=44) and those in their thirties accounting for 41.2% (n=49) of the participants; in addition, 14 (11.8%) participants were in their forties, 4 (3.4%) were in their fifties, and 8 (6.7%) were aged 60 years or above, illustrating that the majority of participants (n=93, 78.2%) were aged 20-39 years. This age distribution mirrored the pronounced involvement of the younger demographic in the metaverse. Gender representation was nearly balanced, with 61 (51.3%) male and 58 (48.7%) female. In terms of geographical distribution, 51 (42.9%) participants were from Seoul, the campaign’s host city, and 68 (57.1%) were from other regions. Regarding disease status, 79 (66.4%) were individuals from the general population without any direct association to the disease or caregiving roles, 33 (27.7%) were family members and caregivers of patients with prostate cancer, and 7 (5.9%) were patients with prostate cancer. With respect to the prostate cancer stage, 3 (42.8%) patients had stage 2 disease, while 2 (28.6%) patients each had stage 3 or 4 disease. Regarding the metaverse experience, 64 (53.8%) participants had prior experience with the metaverse, while 55 46.2%) had not engaged with such virtual environments before (Table 1).

**Table 1:** Participant demographics and characteristics (N=119).

Characteristics		Participants, n (%)
**Age (years)**		
	≤9	0
	20-29	44 (37.0)
	30-39	49 (41.2)
	40-49	14 (11.8)
	50-59	4 (3.4)
	>60	8 (6.7)
**Gender**		
	Male	61 (51.3)
	Female	58 (48.7)
**Patient status**		
	General population	79 (66.4)
	Caregivers	33 (27.7)
	Patients with stage 1 prostate cancer	0
	Patients with stage 2 prostate cancer	3 (2.5)
	Patients with stage 3 prostate cancer	2 (1.7)
	Patients with stage 4 prostate cancer	2 (1.7)
**Region**		
	Seoul	51 (42.9)
	Other cities	68 (57.1)
**The metaverse experience**		
	Yes	64 (53.8)
	No	55 (46.2)

### Confirmatory Factor Analysis

As shown in Table 2, CFA demonstrated an acceptable fit across all indices (*χ*²_71_=1.79, CFI=0.953, TLI=0.940, RMSEA=0.082, SRMR=0.044). These fit indices met the commonly accepted thresholds (CFI/TLI>0.90, RMSEA<0.08, SRMR<0.08), supporting the adequacy of the Understanding the Prostate Cancer Metaverse event satisfaction questionnaire. To further evaluate the reliability and validity of the constructions, CR and AVE values were calculated (Table 3). The CR values ranged from 0.825 to 0.905, indicating high internal consistency. Similarly, the AVE values ranged from 0.575 to 0.760, exceeding the recommended threshold of 0.50 and confirming convergent validity. The distribution of standardized factor loadings further supported convergent validity, with values ranging from 0.60 to 0.88. Regarding discriminant validity, the squared correlations between the constructs were compared to the AVE values. Since the AVE values were greater than the squared correlations, discriminant validity was established.

**Table 2:** Results of CFA^a^.

Fit index	Satisfaction questionnaire	Criterion
*χ*² (*df*)	127.287 (71)	—^b^
*χ*²/*df*	1.79	<3
*P* value	<0.001	>.05
CFI^c^	0.953	>0.95
TLI^d^	0.940	>0.95
RMSEA^e^	0.082	<0.08
SRMR^f^	0.044	<0.08
CR^g^	0.82-0.91	>0.70
AVE^h^	0.65-0.76	>0.50

^a^CFA: confirmatory factor analysis.

^b^Not applicable.

^c^CFI: comparative fit index.

^d^TLI: Tucker-Lewis index.

^e^RMSEA: root mean square error of approximation.

^f^SRMR: standardized root mean square residual.

^g^CR: composite reliability.

^h^AVE: average variance extracted.

**Table 3:** CFA^a^ results for the questionnaire.

Subscale and items		Standard factor loading	Squared multiple correlation	AVE^b^		CR^c^	
**Educational satisfaction**					0.654		0.883
	I believe the event in the metaverse was conducted smoothly.	0.801	0.642	—^d^		—	
	The event was useful for acquiring information about prostate cancer.	0.835	0.697	—		—	
	The metaverse-based event was well organized, facilitating an easy understanding of prostate cancer information.	0.806	0.650	—		—	
	The metaverse-based event was helpful in learning about information related to prostate cancer that I was curious about.	0.792	0.627	—		—	
**Psychological satisfaction**					0.575		0.843
	Participating in the event was engaging and enjoyable.	0.767	0.588	—		—	
	The metaverse-based event was more convenient than participating in offline face-to-face events or Zoom video lectures.	0.668	0.447	—		—	
	Participating in the event through an avatar in the metaverse felt more comfortable than attending in-person events or events via video.	0.822	0.676	—		—	
	I believe that the metaverse-based event allowed for free communication with other participants and lecturers.	0.769	0.591	—		—	
**Overall satisfaction**					0.760		0.905
	I am generally satisfied with the metaverse-based event.	0.860	0.739	—		—	
	I am willing to participate in a metaverse-based event again.	0.880	0.774	—		—	
	I would actively recommend a metaverse-based event to others.	0.876	0.768	—		—	
**Awareness**					0.611		0.825
	I believe that my experience participating in the Understanding the Prostate Cancer Metaverse event has increased my understanding of the metaverse world.	0.752	0.565	—		—	
	I think that there should be more events like the Understanding the Prostate Cancer Metaverse event that use metaverse platforms for disease awareness.	0.798	0.636	—		—	
	I would like to participate in other disease awareness events using metaverse platforms besides the Understanding the Prostate Cancer Metaverse event.	0.795	0.632	—		—	

^a^CFA: confirmatory factor analysis.

^b^AVE: average variance extracted.

^c^CR: composite reliability.

^d^Not applicable.

### Questionnaire Outcomes

The results of our survey with 119 participants showed that participants reported being overall satisfied, as measured using a 5-point Likert scale (Multimedia Appendix 4). Among the 4 categories, educational satisfaction was rated the highest on average (mean 4.17, SD 0.65), followed by overall satisfaction (mean 4.12, SD 0.72), awareness (mean 4.09, SD 0.72), and psychological satisfaction (mean 4.06, SD 0.70). Analysis of responses with scores of 4 or higher (“agree” or “strongly agree”) revealed that based on the proportion of such responses in each scale, 82.8% (394/476) of responses in the educational aspect were rated 4 or higher, followed by 76.6% (365/476) in the psychological aspect, 81% (289/357) in the overall satisfaction aspect, and 80.4% (287/357) in the awareness aspect. Further analysis of each subcategory revealed that the item regarding the benefit of gaining information about prostate cancer received the highest agreement within the educational aspect (mean 4.25, SD 0.716), whereas the item concerning the smooth progression of the event received relatively lower agreement (mean 4.01, SD 0.818). In terms of psychological aspects, the item stating that participating in the event was interesting and enjoyable received the most agreement (mean 4.13, SD 0.791), while the item about free communication with other participants and lecturers was least agreed upon (mean 3.97, SD 0.863). Overall, the item expressing willingness to participate in the metaverse event again received the most agreement (mean 4.18, SD 0.759), and the item about actively recommending it to others received the least agreement (mean 4.08, SD 0.787). In the cognitive aspect, the item expressing a desire to participate in other disease awareness events using the metaverse platform received the most agreement (mean 4.18, SD 0.755), while the item about enhancing understanding of the metaverse world elicited the lowest agreement (mean 3.96, SD 0.942).

The mean educational satisfaction score was 4.17 (SD 0.65), the mean psychological satisfaction score was 4.06 (SD 0.70), the mean overall satisfaction score was 4.12 (SD 0.72), the mean awareness score was 4.09 (SD 0.72), and the mean total scale score was 4.11 (SD 0.62), reflecting the positivity of the participants’ experiences (Table 4).

**Table 4:** Reliability of the survey instrument.

Subscale		Number of items, n (%)	Mean (SD)	Cronbach α	Cronbach α if the item is dropped
Total scale (all items)		14 (100)	4.11 (0.62))	0.948	—^a^
**Survey subscales**					
	Educational satisfaction	4 (28.6)	4.17 (0.65)	0.881	0.938
	Psychological satisfaction	4 (28.6)	4.06 (0.70)	0.842	0.934
	Overall satisfaction	3 (21.4)	4.12 (0.72)	0.903	0.928
	Awareness	3 (21.4)	4.09 (0.72)	0.820	0.935

^a^Not applicable.

Spearman correlation analysis was conducted to investigate the relationship between the 3 Likert items measuring the satisfaction with and recognition of metaverse events (Table 5). Educational satisfaction showed a moderate positive correlation with psychological satisfaction (ρ=0.674, *P*<.001) and overall satisfaction (ρ=0.657, *P*<.001). Moreover, a high positive correlation was observed between psychological satisfaction and overall satisfaction (ρ=0.766, *P*<.001). Additionally, awareness demonstrated a high positive correlation with educational satisfaction (ρ=0.708, *P*<.001), psychological satisfaction (ρ=0.786, *P*<.001), and overall satisfaction (ρ=0.786, *P*<.001).

**Table 5:** Spearman correlations between subscales (N=119).

Subscale and variables		Educational satisfaction	Psychological satisfaction	General satisfaction	Awareness
**Educational satisfaction**					
	Spearman correlation coefficient (ρ)	1.000	0.674^a^	0.657^a^	0.708^a^
	*P* value	—^b^	<.001	<.001	<.001
**Psychological satisfaction**					
	ρ^a^	0.674^a^	1.000	0.766^a^	0.786^a^
	*P* value	<.001	—	<.001	<.001
**General satisfaction**					
	ρ^a^	0.657^a^	0.766^a^	1.000	0.786^a^
	*P* value	<.001	<.001	—	<.001
**Awareness**					
	ρ^a^	0.708^a^	0.786^a^	0.786^a^	1.000
	*P* value	<.001	<.001	<.001	—

^a^The correlation was significant at a significance level of .01 (2-tailed).

^b^Not applicable.

### Subgroup Analysis

Satisfaction and awareness regarding the Understanding the Prostate Cancer Metaverse event were assessed across groups differentiated by age, gender, patient status, geographical location, and the metaverse experience using the Kruskal-Wallis rank sum test and the Mann-Whitney U test (Table 6). Age groups were categorized as twenties, thirties, and ≥40 years. The psychological satisfaction score (median 4.0, IQR 3.50-4.63, vs median 4.50, IQR 4.0-4.56; *P*<.009) and the overall satisfaction score (median 4.0, IQR 3.67-4.83, vs median 4.33, IQR 4.0-4.67; *P*<.025) showed significant differences between the general population group and patients or caregivers, with patients or caregivers reporting higher scores. Significant differences in responses were also noted in awareness scores. Patients or caregivers exhibited higher awareness scores compared to the general population (median 4.0, IQR 3.33-4.33, vs median 4.67, IQR 4.0-4.67; *P*<.005). Furthermore, the Kruskal-Wallis test revealed significant differences in psychological satisfaction among the different age groups, and subsequent post hoc tests with Bonferroni correction identified that individuals in their thirties reported significantly higher psychological satisfaction scores compared to those aged 40 years or older (median 4.25, IQR 3.67-5.0, vs median 3.75, IQR 3.50-4.25; *P*<.041).

**Table 6:** Comparison of group significance for scores by age, gender, patient status, region, and the metaverse experience.

Variable			Subscale, median (IQR)						
			Educational satisfaction		Psychological satisfaction		Overall satisfaction		Awareness
**Age (years)**									
	<30	4.13 (3.75-4.50)		4.13 (3.75-4.50)		4.33 (3.67-4.67)		4.0 (3.67-4.67)	
	30-39	4.25 (4.0-5.0)		4.25 (4.0-4.75)		4.0 (4.0-5.0)		4.33 (3.67-5.0)	
	≥40	4.00 (3.75-4.50)		3.75 (3.50-4.25)		4.0 (3.67-4.33)		4.0 (3.33-4.33)	
	*P* value^a^	.142		.041		.388		.265	
**Gender**									
	Male	4.25 (4.0-4.50)		4.25 (3.75-4.50)		4.0 (3.67-4.67)		4.0 (4.0-4.67)	
	Female	4.0 (3.75-4.94)		4.0(3.56-4.75)		4.0 (3.67-5.0)		4.0(3.42-4.67)	
	*P* value^b^	.753		.589		.946		.510	
**Patient status**									
	Irrelevance	4.0 (3.75-4.75)		4.0 (3.50-4.63)		4.0 (3.67-4.83)		4.0 (3.33-4.33)	
	Relevance	4.25 (4.0-4.75)		4.50 (4.0-4.56)		4.33 (4.0-4.67)		4.67 (4.0-4.67)	
	*P* value^b^	.072		.009		.025		.005	
**Region**									
	Seoul	4.25 (3.75-4.88)		4.0 (3.50-4.75)		4.0 (3.50-4.83)		4.0 (3.33-4.67)	
	Other cities	4.25 (3.94-4.75)		4.25 (3.75-4.50)		4.17 (4.0-4.67)		4.0 (4.0-4.67)	
	*P* value^b^	.769		.596		.227		.181	
**Metaverse experience**									
	Yes	4.25 (3.75-4.81)		4.0 (3.69-4.56)		4.17 (3.67-4.67)		4.0 (3.59-4.67)	
	No	4.0 (3.38-4.63)		4.03 (3.75-4.63)		4.0 (4.0-5.0)		4.0 (3.67-4.67)	
	*P* value^b^	.573		.695		.784		.795	

^a^Kruskal-wallis rank sum test.

^b^Mann-Whitney U test.

To compare the general characteristics of patient-related groups and the general population group, a chi-square test was performed. Significant distribution variations were observed in age (*P*=.001), gender (*P*=.001), and residential area (*P*=.044), whereas differences in the metaverse experience (*P*=.172) were not statistically significant (Table 7).

When participants were asked about their subjective satisfaction with the event, the most common comments included “uniqueness of the metaverse platform,” “accessibility and convenience,” “variety of prostate cancer–related content,” and “real-time face-to-face interactions to answer questions.” Disappointments included ”complexity of the metaverse platform“ and ”incomplete implementation of the metaverse.“

The content that was most appreciated (“best content”) included real-time Q&A sessions about prostate cancer by 27 (22.7%) participants, followed by the introduction of the Blue Ribbon Campaign by 26 (21.9%) participants, videos on prostate cancer treatment by 22 (18.5%) participants, videos on prostate cancer diagnosis by 19 (16%) participants, videos on postoperative care for prostate cancer by 14 (11.8%) participants, and overview videos on prostate cancer by 11 (9.2%) participants.

In terms of preferred formats for events and lectures, the metaverse was favored by 48 (40.3%) participants, followed by online video conferences by 40 (33.6%) participants and offline face-to-face meetings by 31 (26.1%) participants, with no significant difference in response rates. Offline face-to-face communication was considered the most effective by 55 (46.2%) participants; however, for “material sharing,” 62 (52.1%) participants found the use of the metaverse to be the most effective, and for “information dissemination,” this method was preferred by 51 (42.9%) participants.

Regarding topics of interest for future prostate cancer–related events and lectures, participants emphasized the need for information about prostate cancer, prostate cancer surgery and management, cases of patients with prostate cancer, and issues relevant to the families and caregivers of patients with prostate cancer. Especially for patients and their families, practical information that can be applied to daily life is of great interest. This includes topics such as the daily routines of patients with cancer and precautions for caregivers.

**Table 7:** General characteristics of patient-related groups and the general population group.

Characteristics			Participants (N=119)					*χ*² (*df*)		*P* value
			General population (n=79), n (%)		Patients and caregivers (n=40), n (%)					
**Age (years)**								13.815 (2)		.001
	<30	27 (34.2)		17 (42.5)		—^a^			—	
	30-39	41 (51.9)		8 (20.0)		—			—	
	≥40	11 (13.9)		15 (37.5)		—			—	
**Gender**								10.879 (1)		.001
	Male	32 (40.5)		29 (72.5)		—			—	
	Female	47 (59.5)		11 (27.5)		—			—	
**Region**								4.067 (1)		.044
	Seoul	39 (49.4)		12 (30.0)		—			—	
	Other cities	40 (50.6)		28 (70.0)		—			—	
**The metaverse experience**										
	Yes	46 (58.2)		18 (45.0)		1.8693 (1)			.172	
	No	33 (41.8)		22 (55.0)		—			—	

^a^Not applicable.

## Discussion

### Principal Findings

This is the first study to investigate whether incorporating the metaverse platform into a prostate cancer campaign is effective in raising awareness among participants and whether metaverse events are more satisfying to participants than face-to-face methods. The study focused on prostate cancer because this cancer type requires different therapeutic approaches, including active surveillance, surgery, and radiation therapy, depending on the location and severity of the disease, and the choice of treatment is based on a combination of the patient’s age, underlying medical conditions, life expectancy, general health, preferences, and side effects of treatment methods [3,25]. Therefore, it is crucial to offer patients objective and comprehensible information and education regarding the diagnosis and treatment process. Educational counseling can assist patients in comprehending their condition and taking an active role in treatment decisions. Health care professionals have a significant responsibility to clarify the diverse treatment choices that are available and support patients with the challenges they encounter in prostate cancer treatment [26,27]. Additionally, as prostate cancer has a hereditary component [28], we used the metaverse to provide valuable information for caregivers, making it accessible to anyone interested in prostate cancer, not just patients and their caregivers. Participants reported high satisfaction with educational and psychological aspects of the event, indicating positive reactions to the novelty, accessibility, and convenience of the metaverse platform. There was a difference in satisfaction between the general population group versus patients and caregivers, likely due to the event’s content being more directly relevant to patients with prostate cancer and their families than the general population group. Given the broad age of onset of the disease, participants may perceive the same platform differently based on their characteristics. Previous studies have suggested that participants became interested in computers and the internet, which they had not used before, due to their experience with websites [29]. This highlights the importance of exposing people to simple interfaces initially to spark their interest and facilitate adaptation. Additionally, other studies have emphasized the need for education and understanding of the concept of the metaverse itself [30]. Future endeavors should prioritize simplifying the interface rather than implementing multiple functional elements of the metaverse. Developing a metaverse platform that includes information and content related to prostate cancer will be valuable to patients with prostate cancer and their caregivers.

### Strengths and Limitations

A strength of our study is the high internet usage rate in Korea, with 93% of the total population using the internet. Additionally, 94% of people in their sixties and 54.7% of people in their seventies or older use the internet. Furthermore, the penetration rate of mobile devices has reached 92%. Although still in the early stages of introduction, the usage rates of artificial intelligence services and the metaverse are reported to be 42.4% and 11.0%, respectively [31]. This high level of digital awareness, based on the penetration rate and digital literacy, means that conditions for metaverse campaigns in Korea are favorable. Furthermore, the side effects of prostate cancer, such as urinary incontinence, which can occur after prostate cancer surgery, can lead to the use of diapers or cause difficulties in social participation [32]. Measuring sensitive issues, such as erectile dysfunction, accurately is challenging, and discussing them can be uncomfortable [33]. A previous study reported that men who have limited access to medical services and experience social isolation are at a higher risk for both prostate cancer development and death due to prostate cancer [34]. This suggests that the metaverse can play an important role in providing access to needed information, increasing awareness, and improving management without physical limitations.

However, it is important to note the limitations of this study. One limitation concerns the demographic composition of the participant sample. The majority of participants were in their twenties and thirties, and only 5.9% were actual patients with prostate cancer. Although this limits the sample’s representativeness with respect to patients with prostate cancer, the presence of caregivers and family members, who accounted for 27.7% of the participants, provides an important complementary perspective. Younger participants and caregivers can serve as effective intermediaries in disseminating digital health information within families, particularly in contexts where digital literacy varies across generations [29,35]. These individuals also play a significant role in medical decision-making and health communication, and their perceptions offer valuable insights into the development of patient-centered educational strategies [36,37]. Furthermore, the inclusion of the general population in such campaigns may hold long-term value by fostering early awareness, which could positively influence preventive health behaviors and timely cancer screening as these individuals age [38,39]. Considering these points, future research should aim to recruit a more representative sample focusing on patients with prostate cancer or include a broader and more diverse range of patients across different ages and backgrounds.

Another limitation of this study is its exclusive reliance on self-reported satisfaction and awareness data, collected immediately after the intervention using Likert scale items. Although the findings indicated high satisfaction and perceived awareness, such immediate postevent assessments are inherently vulnerable to social desirability bias and may reflect short-term novelty effects rather than genuine cognitive or behavioral change. Moreover, the term ”satisfaction“ was not clearly defined, a limitation previously noted in related literature, and the questionnaire may not have captured the full spectrum of participants’ experiences [40,41]. Additionally, the survey may not have comprehensively covered a broad range of aspects. To adequately address these points, future studies should use more detailed and multidimensional questionnaires to identify specific factors beyond satisfaction before and after participation. This could help increase the depth and accuracy of the research, leading to generalizable results and expanding the reliability and applicability of the study [42,43]. Objective assessments, such as pre- and postintervention knowledge tests or validated prostate cancer knowledge scales, would enable a more rigorous evaluation of educational effectiveness [44]. Furthermore, behavioral follow-up, such as tracking whether participants pursued additional information or shared campaign content with others, could yield valuable insights into the lasting impact of the intervention [45,46]. Integrating qualitative methods, such as interviews or focus groups, would enhance the study’s depth by capturing participants’ subjective experiences, motivations, and potential changes in health communication behavior that may not be reflected in quantitative measures alone.

### Conclusion

This research examined the potential of the metaverse to enhance prostate cancer awareness campaigns. The findings indicate that education and information provision through the metaverse has a positive impact on patients, caregivers, and members of the general population. Furthermore, the results demonstrate that demographic factors influence the perceived effectiveness of metaverse-based health interventions. This study underscores the broader potential of virtual health education in enhancing patient engagement, improving information accessibility, and strengthening health care communication. The findings suggest that the metaverse could significantly enhance awareness and participation in prostate cancer treatment and may also contribute to advancements in other important areas of medical education and information provision.

## Appendix

Multimedia Appendix 1 Translated survey questionnaire for participants.

Multimedia Appendix 2 The number of questions answered by participants across different questions. (Total =119).

Multimedia Appendix 3 Consent for survey participation and data use.

Multimedia Appendix 4 Descriptive statistics and distribution of 5-point Likert scale responses (N=119).

## Abbreviations

AVE: average variance extracted
CFA: confirmatory factor analysis
CFI: comparative fit index
CR: composite reliability
Q&A: question and answer
RMSEA: root mean square error of approximation
SRMR: standardized root mean square residual
TLI: Tucker-Lewis index

## Data Availability

The datasets generated and analyzed during this study are not publicly available due to ethical restrictions but are available from the corresponding author upon reasonable request.
