# Supplementary material for: Analyzing Usage of the Metaverse by Associations of Patients With Prostate Cancer During the 2023 Blue Ribbon Campaign: Cross-Sectional Survey Study
Source: J Med Internet Res. 2025 May 13;27:e63030. doi: 10.2196/63030 (PMC12117273; doi:10.2196/63030)
Supplement: Multimedia Appendix 1 [file jmir_v27i1e63030_app1.docx]

**Appendix 1: Translated survey questionnaire for participants.**

| **2023 Prostate Cancer Awareness Metaverse Event Satisfaction Survey** |
| --- |

| **Screener Questions** |
| --- |

**SQ1. What is your age group? (Based on international age)**

| 1. 10~19 years | 1. 20~29 years | 1. 30~39 years |
| --- | --- | --- |
| 1. 40~49 years | 1. 50~59 years | 1. 60 years or older |

**SQ2. Where do you reside?**

| 1. Seoul | 1. Gyeonggi | 1. Incheon | 1. Daejeon | 1. Gwangju | 1. Daegu |
| --- | --- | --- | --- | --- | --- |
| 1. Ulsan | 1. Sejong | 1. Busan | 1. Gangwon | 1. North Chungcheong | 1. South Chungcheong |
| 1. North Jeolla | 1. South Jeolla | 1. North Gyeongsang | 1. South Gyeongsang | 1. Jeju |  |

**SQ3. What is your gender?**

1. Male (Go to SQ4)
2. Female (Go to SQ5)

**SQ4. Have you ever been diagnosed with prostate cancer?**

1. Yes (Go to SQ6)
2. No (Go to SQ5)

**SQ5. Are you a family member or caregiver of a prostate cancer patient?**

1. Yes (Go to Section A)
2. No (Go to Section A)

**SQ6. What was the stage of your prostate cancer at the time of initial diagnosis?**

1. Stage 1 (A tumor that cannot be felt through a digital rectal exam and is not visible on imaging)
2. Stage 2 (A tumor confined to the prostate)
3. Stage 3 (A tumor that has grown outside the prostate)
4. Stage 4 (A tumor that has spread to nearby organs such as the bladder or rectum)
5. I don’t know

**SQ7. What treatments did you undergo after your initial prostate cancer diagnosis? (Select all that apply)**

1. Conservative treatment (active surveillance, watchful waiting, etc.)
2. Surgery (radical prostatectomy, transurethral resection of the prostate, orchiectomy, etc.)
3. Radiation therapy
4. Hormone therapy
5. Chemotherapy
6. Other ( )
7. I don’t know

**SQ8. What is your current prostate cancer status?**

1. Conservative treatment (active surveillance, watchful waiting, etc.)
2. Maintaining normal status after surgery
3. Maintaining normal status after radiation therapy alone
4. Maintaining normal status after surgery + radiation therapy
5. Maintaining normal status after surgery + radiation therapy + hormone therapy
6. Maintaining normal status after hormone therapy or hormone therapy + chemotherapy
7. Maintaining normal status after other treatments
8. Currently undergoing treatment

| **Section A. Metaverse Usage Experience** |
| --- |

**A1.** **Have you ever used a metaverse platform before?**

1. Yes (Go to A2)
2. No (Go to Section B)

**A2.** **How frequently do you use metaverse platforms?**

1. 4 or more times per week
2. 1–3 times per week
3. Once per month
4. Once per quarter (every 3 months)
5. Once per half-year (every 6 months)
6. Once per year or less

| **Section B. Satisfaction with the Prostate Cancer Awareness Metaverse Event** |
| --- |

**B1.** **How did you learn about the ‘Prostate Cancer Awareness Metaverse Event’?**

1. Prostate Cancer Patients Association
2. Urologic cancer-related communities (e.g., online forums, social media groups)
3. Korean Urological Oncology Society YouTube channel
4. Online articles
5. Recommendation from an acquaintance
6. Other (__________)

**B2.** **Please rate your satisfaction with the educational aspects of the event.**

| **Statement** | **Strongly disagree** | **Disagree** | **Neutral** | **Agree** | **Strongly agree** |
| --- | --- | --- | --- | --- | --- |
| I believe the event in the metaverse was conducted smoothly. |  |  |  |  |  |
| The event was useful for acquiring information about prostate cancer. |  |  |  |  |  |
| The metaverse-based event was well-organized, facilitating an easy understanding of prostate cancer information. |  |  |  |  |  |
| The metaverse-based event was helpful in learning about information related to prostate cancer that I was curious about. |  |  |  |  |  |

**B3.** **Please rate your satisfaction with the psychological aspects of the event.**

| **Statement** | **Strongly disagree** | **Disagree** | **Neutral** | **Agree** | **Strongly agree** |
| --- | --- | --- | --- | --- | --- |
| Participating in the event was engaging and enjoyable. |  |  |  |  |  |
| The metaverse-based event was more convenient than participating in offline face-to-face events or Zoom video lectures. |  |  |  |  |  |
| Participating in the event through an avatar in the metaverse felt more comfortable than attending in-person or via video. |  |  |  |  |  |
| I believe that the metaverse-based event allowed for free communication with other participants and lecturers. |  |  |  |  |  |

**B4.** **Overall satisfaction with the event**

| **Statement** | **Strongly disagree** | **Disagree** | **Neutral** | **Agree** | **Strongly agree** |
| --- | --- | --- | --- | --- | --- |
| I am generally satisfied with the metaverse-based event. |  |  |  |  |  |
| I am willing to participate in a metaverse-based event again. |  |  |  |  |  |
| I would actively recommend a metaverse-based event to others. |  |  |  |  |  |

**B5.** **What was your favorite content from the ‘Prostate Cancer Awareness Metaverse’ platform?**

1. Introduction to the Blue Ribbon Campaign
2. Prostate Cancer Health Lecture: Overview of Prostate Cancer
3. Prostate Cancer Health Lecture: Diagnosis of Prostate Cancer
4. Prostate Cancer Health Lecture: Treatment of Prostate Cancer
5. Prostate Cancer Health Lecture: Post-Surgical Management
6. Live Q&A on Prostate Cancer
7. Other (__________)

**B6. What did you like most about the event?**

_________________________________________________________

**B7.** **What aspects of the event could be improved?**

_________________________________________________________

| **Section C. Awareness of Metaverse Use in Disease Awareness Programs** |
| --- |

**C1.** **Based on your experience participating in the ‘Prostate Cancer Awareness Metaverse Event,’ please indicate your thoughts on the use of the metaverse.**

| **Statement** | **Strongly disagree** | **Disagree** | **Neutral** | **Agree** | **Strongly agree** |
| --- | --- | --- | --- | --- | --- |
| I believe that my experience participating in the ‘Understanding Prostate Cancer via Metaverse’ has increased my understanding of the metaverse world. |  |  |  |  |  |
| I think that there should be more events like the 'Understanding Prostate Cancer via Metaverse' that utilize metaverse platforms for disease awareness. |  |  |  |  |  |
| I would like to participate in other disease awareness events using metaverse platforms besides the ‘Understanding Prostate Cancer via Metaverse’. |  |  |  |  |  |

**C2.** **Which event/lecture format do you prefer the most? (Select one)**

1. Metaverse-based event/lecture
2. Online video conference event/lecture (Zoom, Teams, etc.)
3. In-person event/lecture
4. Other (__________)

**C3.** **Which format do you think is the most effective for smooth communication?**

1. Metaverse-based event/lecture
2. Online video conference event/lecture (Zoom, Teams, etc.)
3. In-person event/lecture
4. Other (__________)

**C4. Which format do you think is the most effective for sharing materials?**

1. Metaverse-based event/lecture
2. Online video conference event/lecture (Zoom, Teams, etc.)
3. In-person event/lecture
4. Other (__________)

**C5.** **Which format do you think is the most effective for delivering information?**

1. Metaverse-based event/lecture
2. Online video conference event/lecture (Zoom, Teams, etc.)
3. In-person event/lecture
4. Other (__________)

**C6. If a future prostate cancer event/lecture is held, what topics or questions would you like to see covered?**

_________________________________________________________
