# Supplementary material for: Analyzing Usage of the Metaverse by Associations of Patients With Prostate Cancer During the 2023 Blue Ribbon Campaign: Cross-Sectional Survey Study
Source: J Med Internet Res. 2025 May 13;27:e63030. doi: 10.2196/63030 (PMC12117273; doi:10.2196/63030)
Supplement: Multimedia Appendix 2 [file jmir_v27i1e63030_app2.docx]

**Appendix 2. The number of questions answered by participants across different questions. (Total =119)**

| **Item** | **Responses, n(%)** |
| --- | --- |
| **Screener** | |
| SQ1. What is your age group? (Based on international age) | 119(100) |
| SQ2. Where do you reside? | 119(100) |
| SQ3. What is your gender? | 119(100) |
| SQ4. Have you ever been diagnosed with prostate cancer? | 61(51.3) |
| SQ5. Are you a family member or caregiver of a prostate cancer patient? | 112(94.1) |
| SQ6. What was the stage of your prostate cancer at the time of initial diagnosis? | 7(5.9) |
| SQ7. What treatments did you undergo after your initial prostate cancer diagnosis? | 7(5.9) |
| SQ8. What is your current prostate cancer status? | 7(5.9) |
| **Section A. Metaverse Usage Experience** | |
| A1. Have you ever used a metaverse platform before? | 119(100) |
| A2. How frequently do you use metaverse platforms? | 64(53.8) |
| **Section B. Satisfaction with the Prostate Cancer Awareness Metaverse Event** | |
| B1. How did you learn about the ‘Prostate Cancer Awareness Metaverse Event’? | 119(100) |
| B2. Please rate your satisfaction with the educational aspects of the event. |  |
| 1. I believe the event in the metaverse was conducted smoothly. | 119(100) |
| 1. The event was useful for acquiring information about prostate cancer. | 119(100) |
| 1. The metaverse-based event was well-organized, facilitating an easy understanding of prostate cancer information. | 119(100) |
| 1. The metaverse-based event was helpful in learning about information related to prostate cancer that I was curious about. | 119(100) |
| B3. Please rate your satisfaction with the psychological aspects of the event. |  |
| 1. Participating in the event was engaging and enjoyable. | 119(100) |
| 1. The metaverse-based event was more convenient than participating in offline face-to-face events or Zoom video lectures. | 119(100) |
| 1. Participating in the event through an avatar in the metaverse felt more comfortable than attending in-person or via video. | 119(100) |
| 1. I believe that the metaverse-based event allowed for free communication with other participants and lecturers. | 119(100) |
| B4. Please rate your overall satisfaction with the ‘Prostate Cancer Awareness Metaverse Event.’ |  |
| 1. I am generally satisfied with the metaverse-based event. | 119(100) |
| 1. I am willing to participate in a metaverse-based event again. | 119(100) |
| 1. I would actively recommend a metaverse-based event to others. | 119(100) |
| B5. What was your favorite content from the ‘Prostate Cancer Awareness Metaverse’ platform? | 119(100) |
| B6. What did you like most about the event? | 84(70.6) |
| B7. What aspects of the event could be improved? | 76(63.9) |
| **Section C. Perception of Metaverse Use in Disease Awareness Programs** | |
| C1. Based on your experience participating in the ‘Prostate Cancer Awareness Metaverse Event,’ please indicate your thoughts on the use of the metaverse. |  |
| 1. I believe that my experience participating in the ‘Understanding Prostate Cancer via Metaverse’ has increased my understanding of the metaverse world. | 119(100) |
| 1. I think that there should be more events like the 'Understanding Prostate Cancer via Metaverse' that utilize metaverse platforms for disease awareness. | 119(100) |
| 1. I would like to participate in other disease awareness events using metaverse platforms besides the ‘Understanding Prostate Cancer via Metaverse’. | 119(100) |
| C2. Please select your preferred event/lecture format. | 119(100) |
| C3. Which event/lecture format do you think is the most effective for smooth communication? | 119(100) |
| C4. Which event/lecture format do you think is the most effective for sharing materials? | 119(100) |
| C5. Which event/lecture format do you think is the most effective for delivering information? | 119(100) |
