# Supplementary material for: Analyzing Usage of the Metaverse by Associations of Patients With Prostate Cancer During the 2023 Blue Ribbon Campaign: Cross-Sectional Survey Study
Source: J Med Internet Res. 2025 May 13;27:e63030. doi: 10.2196/63030 (PMC12117273; doi:10.2196/63030)
Supplement: Multimedia Appendix 3 [file jmir_v27i1e63030_app3.docx]

**Appendix 3: Consent for survey participation and data use**

| **2023 Prostate Cancer Awareness Metaverse Event Satisfaction Survey** |
| --- |

| **Survey Description** |
| --- |

Thank you for taking the time to participate in this survey. This survey is part of the **Blue Ribbon Campaign** led by the **Korean Urological Foundation** and the **Korean Society of Urological Oncology**, aimed at raising awareness of prostate cancer. Specifically, the purpose of this survey is to collect participants’ feedback on the “**2023 Understanding Prostate Cancer Metaverse Event**.”

The survey consists of **20 questions** and is expected to take approximately **5 to 10 minutes** to complete. Participants who complete the entire survey will be entered into a prize draw to receive **Gift certificates** (KRW 50,000 for 3 winners, KRW 10,000 for 30 winners, to be distributed in October 2023).

All responses will be statistically processed in a manner that prevents individual identification, and the aggregated results will be used solely for the purpose of evaluating and improving the prostate cancer awareness campaign. This study adheres to Article 33 (Confidentiality) of the Statistics Act, and all responses will remain strictly confidential. Your input will be used as valuable reference data.

- **Organizers:** Korean Urological Foundation, Korean Society of Urological Oncology
- **Survey Manager:** Enzyme Health Inc.

| **Do you agree to the collection and use of your personal information?**  ○ Yes, I agree ○ No, I do not agree (survey will end) |
| --- |
