# Supplementary material for: Analyzing Usage of the Metaverse by Associations of Patients With Prostate Cancer During the 2023 Blue Ribbon Campaign: Cross-Sectional Survey Study
Source: J Med Internet Res. 2025 May 13;27:e63030. doi: 10.2196/63030 (PMC12117273; doi:10.2196/63030)
Supplement: Multimedia Appendix 4 [file jmir_v27i1e63030_app4.docx]

**Multimedia Appendix 4.** Descriptive statistics and distribution of 5-point Likert scale responses (N=119).

| Subscale and items | | | Mean (SD) | Frequency (%) |
| --- | --- | --- | --- | --- |
| **Educational satisfaction** | | | 4.17 (0.65) |  |
| I believe the event in the metaverse was conducted smoothly. | | | 4.01 (0.818) |  |
| The event was useful for acquiring information about prostate cancer. | | | 4.24 (0.701) |  |
| The metaverse-based event was well organized, facilitating an easy understanding of prostate cancer information. | | | 4.18 (0.770) |  |
| The metaverse-based event was helpful in learning about information related to prostate cancer that I was curious about. | | | 4.25 (0.716) |  |
| **Psychological satisfaction** | | | 4.06 (0.70) |  |
| Participating in the event was engaging and enjoyable. | | | 4.13 (0.791) |  |
| The metaverse-based event was more convenient than participating in offline face-to-face events or Zoom video lectures. | | | 4.01 (0.859) |  |
| Participating in the event through an avatar in the metaverse felt more comfortable than attending in-person events or events via video. | | | 4.13 (0.879) |  |
| I believe that the metaverse-based event allowed for free communication with other participants and lecturers. | | | 3.97 (0.863) |  |
| **Overall satisfaction** | | | 4.12 (0.72) |  |
| I am generally satisfied with the metaverse-based event. | | | 4.10 (0.807) |  |
| I am willing to participate in a metaverse-based event again. | | | 4.18 (0.759) |  |
| I would actively recommend a metaverse-based event to others. | | | 4.08 (0.787) |  |
| **Awareness** | | | 4.09 (0.72) |  |
| I believe that my experience participating in the Understanding the Prostate Cancer Metaverse event has increased my understanding of the metaverse world. | | | 3.96 (0.942) |  |
| I think that there should be more events like the Understanding the Prostate Cancer Metaverse event that use metaverse platforms for disease awareness. | | | 4.13 (0.798) |  |
| I would like to participate in other disease awareness events using metaverse platforms besides the Understanding the Prostate Cancer Metaverse event. | | | 4.18(0.755) |  |
| ■ 1 = Strongly disagree | ■ 2 = Disagree | ■ 3 = Neutral | ■ 4 = Agree | ■ 5 = Strongly agree |
